# Kynurenines link chronic inflammation to functional decline and physical frailty

**DOI:** 10.1172/jci.insight.136091

**Published:** 2020-08-20

**Authors:** Reyhan Westbrook, Tae Chung, Jacqueline Lovett, Chris Ward, Humberto Joca, Huanle Yang, Mohammed Khadeer, Jing Tian, Qian-Li Xue, Anne Le, Luigi Ferrucci, Ruin Moaddel, Rafa de Cabo, Ahmet Hoke, Jeremy Walston, Peter M. Abadir

**Affiliations:** 1 Division of Geriatric Medicine and Gerontology,; 2 Department of Physical Medicine and Rehabilitation, and; 3 Department of Neurology, Johns Hopkins University School of Medicine, Baltimore, Maryland, USA.; 4 National Institute on Aging, NIH, Baltimore, Maryland, USA.; 5 Department of Orthopedics and Biomedical Engineering and Technology, University of Maryland School of Medicine, Baltimore, Maryland, USA.; 6 Department of Oncology and; 7 Department of Pathology, Johns Hopkins University School of Medicine, Baltimore, Maryland, USA.; 8 Department of Medicine, Kyung Hee University, Seoul, South Korea.

**Keywords:** Aging, Inflammation, Cytokines, Neurodegeneration

## Abstract

Chronic inflammation is associated with physical frailty and functional decline in older adults; however, the molecular mechanisms of this linkage are not understood. A mouse model of chronic inflammation showed reduced motor function and partial denervation at the neuromuscular junction. Metabolomic profiling of these mice and further validation in frail human subjects showed significant dysregulation in the tryptophan degradation pathway, including decreased tryptophan and serotonin, and increased levels of some neurotoxic kynurenines. In humans, kynurenine strongly correlated with age, frailty status, TNF-αR_1_ and IL-6, weaker grip strength, and slower walking speed. To study the effects of elevated neurotoxic kynurenines on motor neuronal cell viability and axonal degeneration, we used motor neuronal cells treated with 3-hydroxykynurenine and quinolinic acid and observed neurite degeneration in a dose-dependent manner and potentiation of toxicity between 3-hydroxykynurenine and quinolinic acid. These results suggest that kynurenines mediate neuromuscular dysfunction associated with chronic inflammation and aging.

## Abstract 2

Tryptophan-related toxic metabolites known as kynurenines are altered with chronic inflammation, which damages nerves in aged and frail mice and humans.

## Graphical abstract

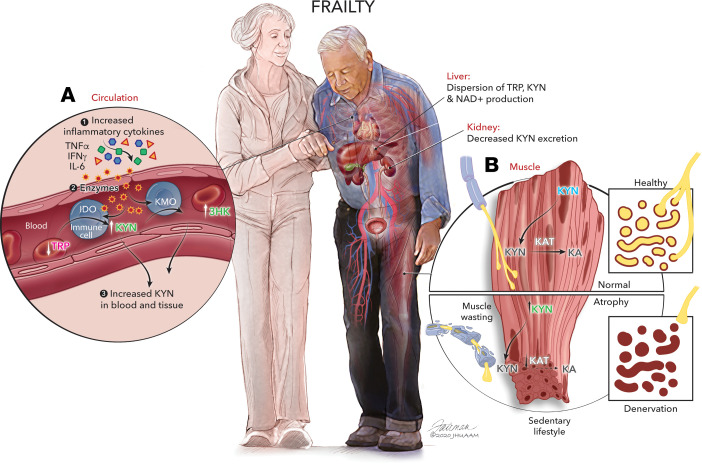

## Introduction

Chronic inflammation, as measured by persistently elevated serum levels of inflammatory cytokines, has long been hypothesized to play a role in the development of multiple adverse conditions related to aging, including disability, physical frailty, mild cognitive impairment, and early mortality (1–3). Furthermore, inflammatory cytokine levels independently correlate with declines in muscle mass and neuromuscular function, including grip strength, and mobility scores (4). In addition, aging is known to affect all components of the neuromuscular system, including brain, spinal neurons, peripheral axons, and muscle tissues, which contribute to functional decline in older adults. Although these findings suggest a potential etiologic role for chronic inflammation in frailty and functional decline, the mechanism that connects inflammation to these conditions has not been elucidated.

To study the biological connections between chronic inflammation and functional decline, we used the IL-10 B6.129P2-*IL 10^tm1Cgn^*/J (IL-10^tm^) mouse (5–10). The lack of the antiinflammatory cytokine IL-10 in this mouse contributes to increased expression of NF-κB–induced inflammatory mediators including IL-1β, TNF-α, IFN-γ, IL-6, and chemokine ligand-1 (11–13). With chronic systemic activation of inflammatory pathways early in life, these mice undergo accelerated changes consistent with late-life decline, including decreased muscle mass, muscle and bone weakness, and altered skeletal muscle gene profile (5–10, 14). Thus, this mouse model could be used to help identify metabolomic signatures of chronic inflammation–related changes that could help inform the mechanisms that connect chronic inflammation to physical frailty and functional decline in older adults.

In order to pinpoint specific biological pathways that connect chronic inflammation to functional decline and physical frailty, we used a targeted metabolomic platform to identify categories or signatures of differences in metabolite levels. We first profiled the plasma of young, middle-aged, and old-aged IL-10^tm^ and control mice and saw prominent and consistent alterations in the tryptophan (TRP) degradation pathway, which became stronger as the mice aged. Specifically, we saw decreased TRP and serotonin (5HT) and concomitantly increased kynurenine (KYN) in middle-aged IL-10^tm^ mice relative to control mice and intensification of these changes in old-aged IL-10^tm^ mice relative to age-matched controls. We then validated these results in serum from a cross-sectional cohort of young, nonfrail, and frail adults and again saw increased TRP degradation pathway activation with age and frailty status. We followed up on these findings with a series of experimental measures in both mice and human subjects to confirm and extend initial metabolomic findings described below.

TRP degradation occurs primarily through the production of KYNs in mammals and, thus, is sometimes known as the KYN pathway (a small amount of TRP is converted to 5HT and melatonin, which are not part of the KYN pathway). Proinflammatory cytokines activate indolamine 2,3 dioxygenase (IDO), which controls the rate-limiting step of the pathway, the conversion of TRP to KYN (15, 16), and KYN monooxygenase (KMO), which converts KYN to 3HK (17). The enzyme rate–limiting step of the KYN pathway can be activated by inflammatory cytokines and produce molecular intermediates, which are known to be cytotoxic and neurotoxic (reviewed in ref. 18). Based on the results from metabolomic profiling in mice and humans, we hypothesized that downstream neurotoxic and cytotoxic metabolites from the KYN pathway were increased in the frail individuals and that these toxic metabolites cause damage to the peripheral neuromuscular system, eventually leading to frailty and functional decline. To explore this hypothesis, we performed a detailed analysis of the downstream metabolites of the KYN pathway and assessed how levels of these metabolites known as KYNs are altered with age and frailty in humans. We also assessed the neuromuscular function of the IL-10^tm^ mouse and measured both the presynaptic and postsynaptic components of the neuromuscular junction (NMJ) in quadriceps from aged IL-10^tm^ mice. Finally, we evaluated the toxicity of KYNs identified from our metabolomic measurements on a motor neuronal cell line by measuring axonal degeneration and cell viability.

## Results

### Aged IL-10^tm^ mice show reduced motor function and partial denervation of the NMJ.

**Figure 1:**
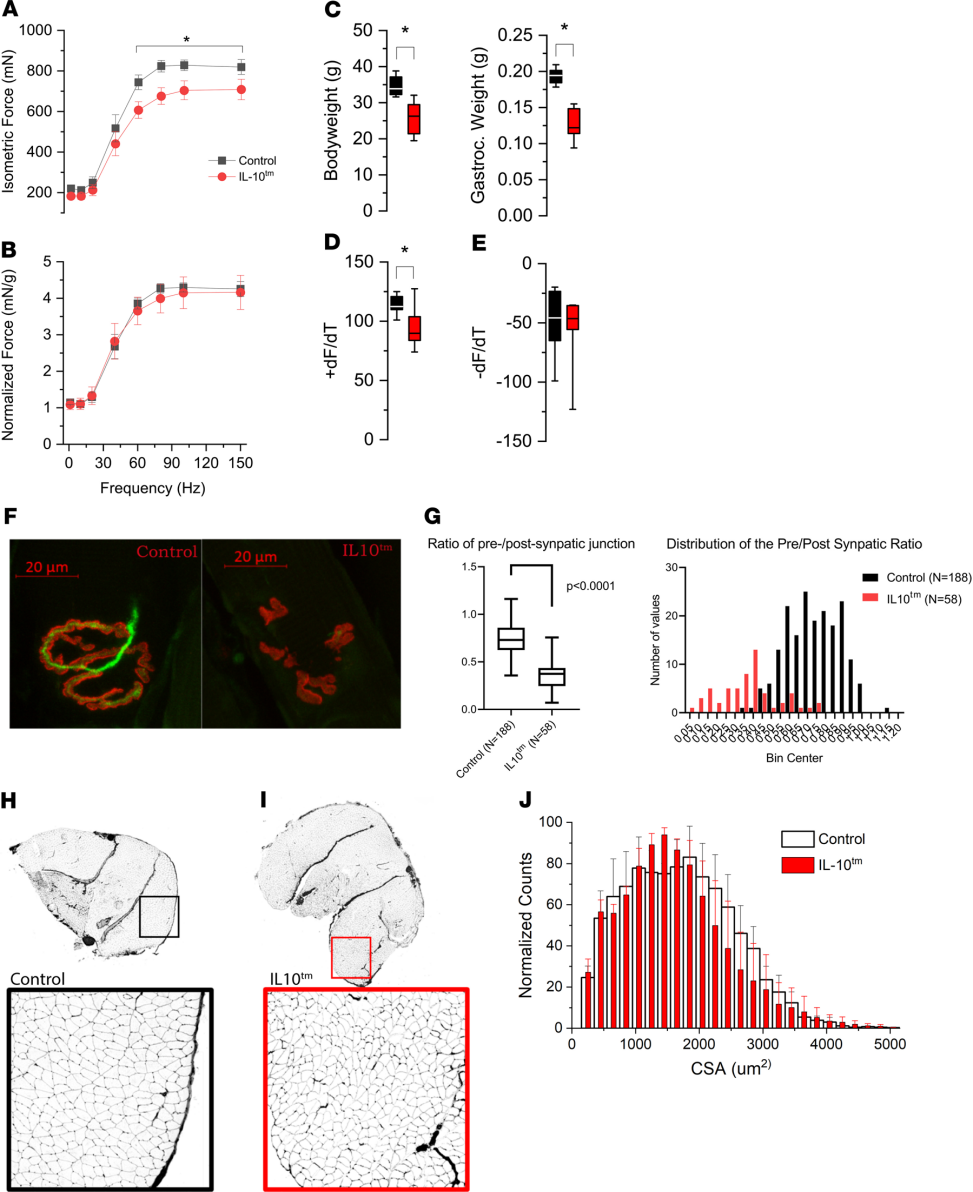
Aged IL-10^tm^ mice show reduced motor function and partial denervation of the neuromuscular junction. (**A**) Isometric force versus in vivo stimulation frequency of the gastrocnemius muscle showed significantly reduced force in IL-10^tm^ mice (aged control, *n* = 7; IL-10^tm^, *n* = 8). (**B**) Force normalized to gastrocnemius weight showed no difference in total force. (**C**) Total body and gastrocnemius weight are lower in IL-10^tm^ mice as compared with those of age-matched control. (**D** and **E**) Kinetics of contraction assessed at 80 Hz showed the maximal rate of contraction (+dF/dt) was significantly lower in the IL-10^tm^ mice (**D**), while the maximal rate of relaxation (–dF/dT) was unchanged (**E**). (**F**) Morphology of the neuromuscular junction (NMJ) was quantified with β-3 tubulin (green) to denote the presynaptic area and bungarotoxin (red) to visualize the postsynaptic area. Representative confocal image of an NMJ in old WT B6 mice (control) reveals the presynaptic neural structure (green) opposed to a prototypical postsynaptic NMJ end-plate. Adjacent is a representative NMJ from old IL-10^tm^ mice showing the absence of presynaptic neural connectivity (green) and disorganized postsynaptic structure (red). (**G**) Semiquantitative analysis of NMJ morphology. The first panel shows the ratio of presynapatic to postsynapatic junctional area is significantly reduced in IL-10^tm^ mice (*P* < 0.0001). The second panel shows the distribution of the ratio between control old B6 mice versus IL-10^tm^ mice. (**H** and **I**) Cross section of whole gastrocnemius muscle with Wheat Germ Agglutinin (WGA) labeling to detect muscle fiber boundaries (fluorescence image inverted for clarity). (**H**) Muscle fibers from old control mice show rounded muscle fibers of variable size. Magnification, 10×. (**I**) Myofibers of IL-10^tm^ mouse are smaller in diameter than control with many being angular in shape. (**J**) Quantification of muscle fiber cross sectional area (CSA) shows a significant (*P* < 0.0001) reduction in IL-10^tm^ mice compared with age matched control.

IL 10^tm^ mice undergo accelerated neuromuscular decline including decreased muscle mass, muscle and bone weakness, and altered skeletal muscle gene profile (5–10, 13, 14, 19, 20). To dissect the association between chronic inflammation and neuromuscular decline with aging, we tested neuromuscular function in aged IL-10^tm^ mice. Using an in vivo assessment of nerve-evoked plantar flexor function, we evaluated the force versus frequency relationship between aged control (*n* = 7) and IL-10^tm^ (*n* = 8) mice. We show that total isometric force was reduced in the IL-10^tm^ at frequencies greater than 60 Hz (Figure 1A). Given that the body weight and gastrocnemius muscle mass were significantly lower in the IL-10^tm^ mice in aged (Figure 1C) but not young (Supplemental Figure 1; supplemental material available online with this article; https://doi.org/10.1172/jci.insight.136091DS1) mice, we normalized the contractility to muscle mass to determine the impact of this loss to the functional deficit (Figure 1B). Here, we show that the loss in muscle mass in the IL-10^tm^ fully accounted for the functional deficits in that model. Further analysis revealed that the maximal rate of tetanic contraction (+dF/dt; assessed at 80 Hz) was significantly lower in the IL-10^tm^ mice (Figure 1D), with no change in the rate of relaxation (Figure 1E).

To investigate whether morphological changes in the muscle fiber or NMJ accompany functional changes in the IL-10^tm^ mouse, we performed histological assessments of each. Using immunofluorescent staining, we quantitated the pre- and postsynaptic areas of NMJ structure in extensor digitorum muscle (EDL) of IL-10^tm^ and control mice. We show reduced presynaptic to postsynaptic coverage in the IL-10^tm^ mice, suggesting increased partial denervation in their muscles (Figure 1, F and G). In cross sections of the gastrocnemius muscle, we show a significant decrease in muscle fiber CSA in IL-10^tm^ mice with the larger fiber population (i.e., 1500–2500 μm^2^) most affected (Figure 1, H–J). Taken together, reduced neuromuscular connectivity in these mice is a likely contributor to the reduction in muscle fiber CSA, muscle mass, and isometric force in IL-10^tm^ mice, suggesting that age-associated neuromuscular decline is facilitated in IL-10^tm^ mice, as compared with WT mice.

### TRP degradation pathway was the most prominently altered pathway in IL-10^tm^ mice across life span.

In order to pinpoint specific biological pathways that connect chronic inflammation to the observed decline in neuromuscular function in these mice, we used a targeted metabolomic platform to identify categories or signatures of differences in metabolite levels. We compared the plasma levels of an array of metabolites from 5 substance classes in young (5 months), middle-aged (12 months,) and old-aged (22 months) IL-10^tm^ and control mice. Our initial profiling identified several metabolomic alterations in the plasma of the proinflammatory IL-10^tm^ mouse in all 3 age groups. After applying the Holm-Bonferroni’s multiple comparisons correction, there were no significantly altered metabolites in the young group, 2 significant differences in the middle-aged group (the KYN/TRP ratio and phosphatidylcholine acyl-alkyl C42:1 [PC ae C42:1; Table 1]), and 5 significantly altered metabolites in the old group (the KYN/TRP ratio, α–amino adipic acid [α-AAA], TRP, sphingomyelin C18:0, and lysophosphatidylcholine acyl C20:4 [lysoPC a C20:4; Table 1]). One of the most prominent and consistent alterations in the IL-10^tm^ mouse was decreased TRP with a concomitant increase in KYN seen in both middle-aged and old-aged IL-10^tm^ mice. All significant metabolite alterations before multiple comparison corrections are available in Supplemental Tables 1–3.

### Metabolomic profiling in humans shows age-related changes in the TRP degradation pathway and arginine metabolism.

We next profiled the plasma metabolome of both older (nonfrail and frail) and younger human subjects to confirm and extend initial findings in the mouse model. We analyzed the effect of age on the serum levels of an array of metabolites from 5 substance classes by comparing these metabolite levels in young (mean age 25.6 years, *n* = 50) and older subjects (mean age = 77.6 years, *n* = 116) (patient demographics in Supplemental Table 4). Using the Holm-Bonferroni’s method of correction for multiple comparisons (with α = 5.0%), 35 (and 6 preconfigured ratios) of the 121 metabolites measured were significantly altered (Table 2).

Interestingly, metabolically linked metabolites from 2 amino acid degradation pathways, TRP and arginine, were among the metabolites that differed most significantly between young and older subjects. Several metabolites from the TRP degradation pathway were significantly altered in older subjects compared with young. These include increased KYN and an increased KYN/TRP ratio. 5HT, another metabolite derived from TRP, was decreased in older subjects. Together, these alterations in TRP-related metabolites indicate that TRP degradation through the KYN pathway was increased in old subjects compared with young (Table 2).

Among metabolites related to arginine metabolism, symmetric dimethyl arginine (SDMA) and asymmetric dimethyl arginine (ADMA) were increased in old subjects compared with young. Additionally, we saw increased SDMA/arginine, citrulline/arginine, and citrulline/ornithine ratios and increased ornithine in older subjects as compared with younger subjects. The global arginine bioavailability ratio (GABR, arginine/[citrulline + ornithine]) was significantly lower in older subjects compared with young (Table 2).

In addition to amino acid metabolites, we saw changes in a number of lipid-related metabolites. Octadecenoylcarnitine (C18:1) and 3 sphingomyelins, SM (OH) C14:1, SM C16:1, and SM (OH) C16:1 were increased in old subjects compared with young. Twenty-five different glycerophospholipids (phosphatidyl cholines and lysophosphatidyl cholines) were altered in older subjects compared with young (Table 2). Average CV for all metabolites can be found in Supplemental Table 5.

### TRP degradation pathway metabolites have strongest correlation with frailty status, walking speed, IL-6, and TNF-αR1.

Given that, within the general population, frail older adults experience increased vulnerability to adverse health outcomes, we stratified our older patient groups based on physical frailty status (frail [average age 79.8, *n* = 33], nonfrail [average age 77, *n* = 83]) (Supplemental Table 6) and used linear regression models to estimate the association between metabolite levels and cytokines (IL-6, IL-1, IFN-γ, TNF-α, and TNF-αR1) (Table 3), walking speed, and grip strength (Table 4). Furthermore, we used logistic modeling to estimate the odds ratio of being frail versus nonfrail in relation to blood levels of each metabolite (Table 4).

After adjusting for age, the KYN/TRP ratio was the most strongly correlated metabolite with TNF-αR1 levels. The KYN/TRP ratio was also strongly correlated with IL-6, IFN-γ, and TNF-α levels (Table 3). The KYN/TRP ratio was the top metabolite measurement associated with frailty status and the top predictor of walking speed (Table 4). Additionally, KYN levels alone were the top predictor of IL-6 levels and significant predictors of IFN-γ, TNF-α, and TNF-αR1 levels (Table 3). TRP level was a significant predictor of TNF-αR1 level (Table 3), a significant risk factor for frailty, and a significant predictor of grip strength and walking speed (Table 4). Adjusting for age, sex, BMI, and systolic and diastolic blood pressure produced similar results.

**Figure 2:**
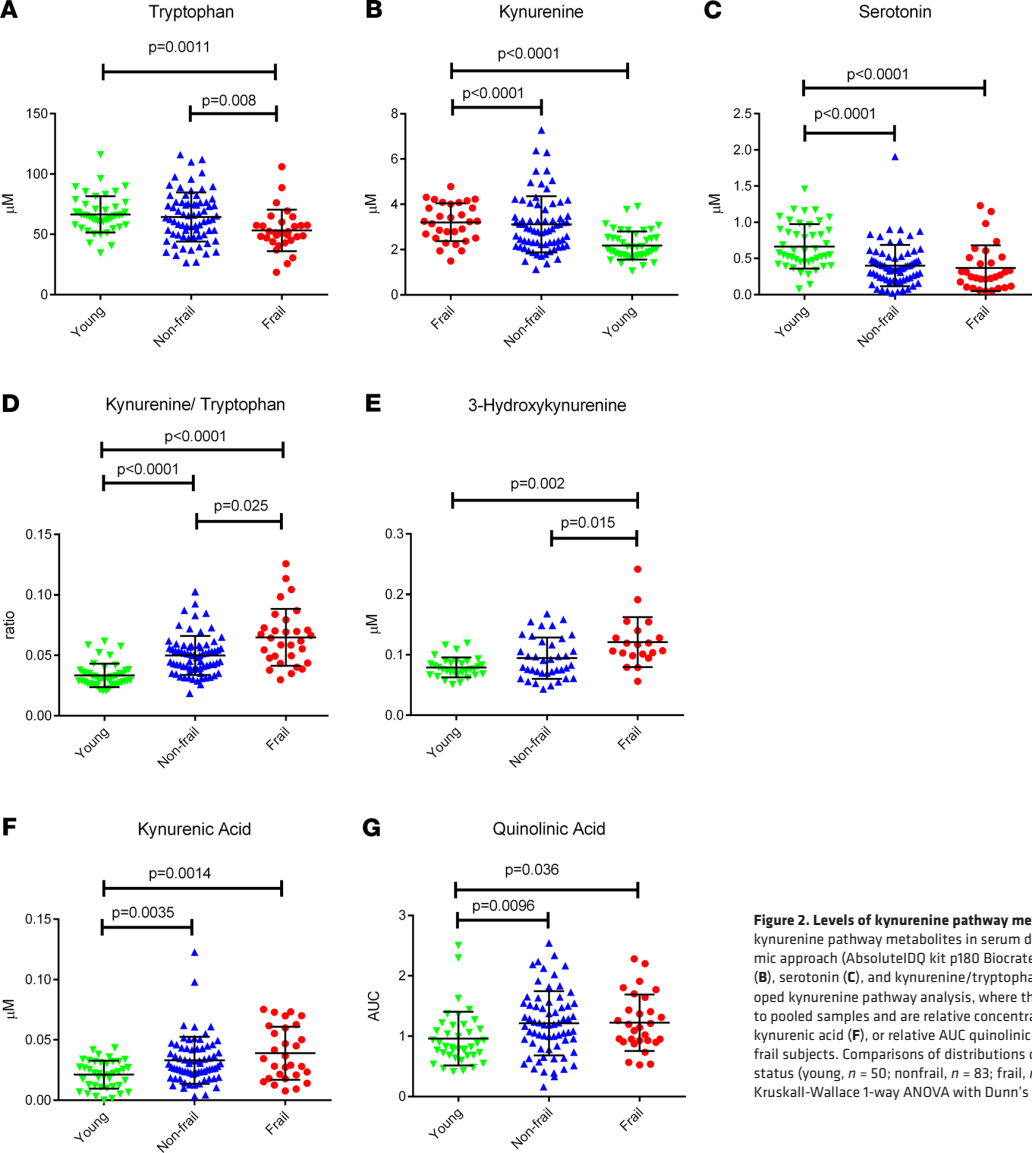
Levels of kynurenine pathway metabolites in serum. (**A–G**) Levels of kynurenine pathway metabolites in serum determined by a targeted metabolomic approach (AbsoluteIDQ kit p180 Biocrates) for tryptophan (**A**), kynurenine (**B**), serotonin (**C**), and kynurenine/tryptophan ratio (**D**) in μM and by the developed kynurenine pathway analysis, where the reported values are normalized to pooled samples and are relative concentrations 3-hydroxykynurenine (**E**), kynurenic acid (**F**), or relative AUC quinolinic acid (**G**) in young, nonfrail, and frail subjects. Comparisons of distributions of metabolites by age and frailty status (young, *n* = 50; nonfrail, *n* = 83; frail, *n* = 33) were conducted using the Kruskall-Wallace 1-way ANOVA with Dunn’s multiple comparisons test.

When we compared the mean levels of TRP-related metabolites between groups, we saw decreased TRP in frail subjects compared with young and nonfrail subjects (Figure 2A). KYN was increased in frail and nonfrail subjects compared with young (Figure 2B). 5HT was decreased in frail and nonfrail subjects compared with young (Figure 2C). Additionally, we saw an increased KYN/TRP ratio in the serum of frail subjects compared with nonfrail and young subjects. This ratio was increased in nonfrail subjects compared with young subjects (Figure 2D).

### Downstream toxic KYN pathway metabolites are altered with frailty.

Our initial results suggested that alterations in the TRP, 5HT, and KYN were significantly linked to age, frailty status, walking speed, and inflammatory cytokine levels. Because the KYN pathway produces several bioactive metabolites that are implicated in a number of disorders and diseases as etiological factors, we systematically profiled other KYN pathway intermediate metabolites including kynurenic acid (KA), 3-hydroxykyn (3HK), xanthurenic acid (XA), quinolinic acid (QA), and PA in the serum of our subject cohort.

3HK was increased in frail compared with both nonfrail and young subjects (Figure 2E). KA and QA were increased in frail and nonfrail subjects compared with young subjects (Figure 2, F and G). XA and PA were not significantly altered between any patient groups (not shown).

Several downstream KYN pathway metabolites significantly correlated with cytokines, functional outcomes, and frailty status, as well. After age and multivariable adjustment, 3HK was significantly correlated with frailty status and walking speed (Supplemental Table 7). KA was significantly correlated with TNF-αR1 levels (Supplemental Table 7).

### Increased production of KYNs is linked to increased inflammation and impaired physical function.

Given the variability in genetic background, physiology, pathophysiology, and diet between subjects in this population, we examined ratios of the different metabolites within the KYN pathway in each individual as a tool to mitigate the impact of between-subject variation of single metabolite levels in order to investigate relationships between KYN metabolites and uncover interesting insights on this pathway in relation to aging and frailty. After age and multivariable adjustment, we observed a trend of increased 3HK relative to several surrounding metabolites (TRP, KYN, XA, and PA) being significantly correlated with frailty status (Supplemental Table 7). Higher TRP relative to downstream metabolites (QA, 3HK, and XA]) was correlated with increased grip strength. Higher TRP relative to downstream metabolites (XA, 3HK, KA, and 5HT) was associated with increased walking speed, while increased 3HK relative to TRP and PA was associated with decreased walking speed (Supplemental Table 7).

Decreased 5HT relative to other KYNs (TRP, KYN, AA, 3HK, and KA) was significantly correlated with increased TNF-α, and various combinations of increased levels of central pathway metabolites (KYN, KA, 3HK, and XA) relative to peripheral pathway metabolites (TRP, 5HT, QA, and PA) were linked with increased cytokine levels (Supplemental Table 7). Together, these data indicate that increased degradation of TRP toward the production of KYNs is linked to increased inflammation and impaired physical function in human subjects.

### 3HK and QA have neurotoxicity on peripheral motor neurons and can potentiate the toxicity in the presence of each other at lower concentrations.

**Figure 3:**
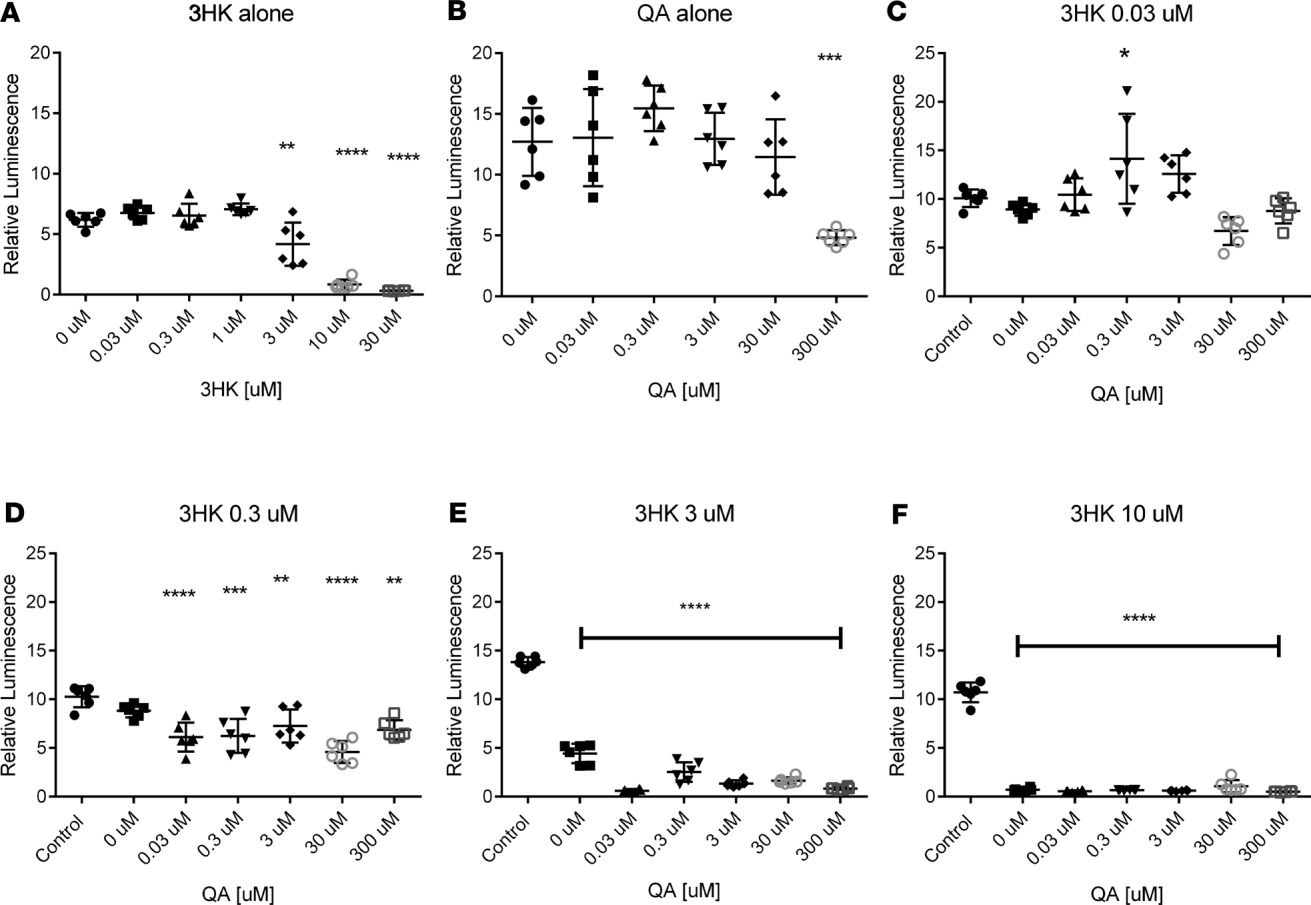
Measurement of ATP levels of MN1 cells under various concentrations of 3HK and QA. (**A** and **B**) When MN1 cells are incubated under 3HK and QA, there is neurotoxicity in a dose-dependent manner. Note that ATP levels significantly drop between 1 and 3 μM of HK and 30 and 300 μM of QA, both of which are supraphysiological concentration in those animals. (**C–F**) When MN1 cells are incubated simultaneously with 3HK and QA, neurotoxicity is potentiated. Data are shown as mean ± SD; *n* = 6 wells, representative of 3 independent experiments. Data were analyzed using 1-way ANOVA, followed by Tukey’s multiple comparisons test (*P < 0.05; **P < 0.005; ***P < 0.0005; ****P < 0.0001, when compared with control).

KYN pathway intermediate metabolites are known to have toxic effects on cerebral neurons (21), but little is known about their effects on the peripheral nervous system. Based on our observation of increased levels of the neurotoxic metabolite 3HK in frail individuals, we tested whether known neurotoxic metabolites 3HK and QA have toxicity on peripheral motor neurons. We used the murine motor neuron cell line MN1 cells and cocultured the cells with 3HK and QA at various concentrations. We measured the amount of ATP in the neurons as a surrogate measure of neurite degeneration, a method previously validated in the laboratory of one of our coauthors (22–24). The reduction in ATP suggests shortening of axonal length, resembling Wallerian-like degeneration. Both 3HK and QA showed neurotoxicity (reduced relative luminescence) with MN1 cells in a dose-dependent manner; for 3HK, toxicity occurs around 1–10 μM, whereas toxicity of QA occurs around a 30–100 μM range (Figure 3, A and B). However, these concentrations are much higher than physiological concentrations found in human brain and peripheral tissues (21). A previous in vivo study showed that 3HK potentiates the toxicity of QA in rat brain, and we hypothesized that the potentiated toxicity also happens on peripheral motor neurons. In fact, toxicity of QA occurred < 0.03 μM in the presence of 3 μM of 3HK, which are close to the concentrations found in human and animals during inflammation or certain conditions that affect kidney or liver function (25, 26) (Figure 3, C–F). These data indicate that increased levels of 3HK seen in frail individuals may have deleterious effects on the motor neuronal system.

## Discussion

Chronic elevations in inflammatory mediators are common in older adults and predict a host of adverse health outcomes, sarcopenia, and physical frailty (27). To date, few specific molecular mechanisms have been identified that connect chronic inflammation to these conditions commonly found in older adults. With the growing global aging population, sarcopenia, physical frailty, and their etiological underpinnings will likely be increasingly relevant to clinicians caring for vulnerable older adults. While some progress has been made in identifying and measuring frailty (28, 29) and predicting mortality (2), little is known about the etiological links between physical frailty and the functional decline observed in frail older adults. Metabolomic measurements focused on a specific biologically relevant pathway as pursued in this study will enable deeper analysis of biological systems in an efficient and high-throughput manner.

In this study, we used metabolomic profiling to identify an amino acid degradation pathway whose activity is altered in response to chronic inflammation in both mice and humans. We integrated this metabolomic data with cytokine and functional measurements from humans, morphological and in vivo functional measurements from mice, as well as in vitro neuronal cell culture data to substantiate evidence that alterations in this molecular pathway not only provide a robust biomarker for frailty, but are likely influencing the course of neuromuscular decline in physical frailty.

We were able to analyze 2 aspects of the circulating metabolites from the mouse and human cohorts in this study: metabolites that changed with age and metabolites that were altered with frailty status. Key findings from the mouse metabolomic study include the elevation of circulating levels of KYNs with aging and chronic inflammation. This increase in KYNs was accompanied with decreased neuromuscular function and loss of integrity at the NMJ. In humans, our metabolomic profiling showed decreased TRP and 5HT — and increased KYN, KYN/TRP ratio, 3HK, KA, and QA — in the serum of our older subjects compared with younger subjects. Those older adults who were also frail had further depletion of TRP and an additional increase in the KYN/TRP ratio and in 3HK compared with nonfrail older adults (summarized in Supplemental Figure 2). The accumulation of KYNs in the blood had a strong correlation with many other covariables, including increased inflammatory cytokines and decreased grip strength and walking speed.

TRP is an essential amino acid and is required for the synthesis of proteins, 5HT, and melatonin, as well as a group of metabolites known as the KYNs, whose metabolic pathway leads to the production of the energy metabolite and cellular cofactor, nicotinamide adenine dinucleotide (NAD^+^) (30) in the de novo pathway. More than 95% of available TRP is degraded through the KYN pathway, with the conversion of TRP to N-formylkyn being the rate-limiting step of the pathway (31). This pivotal step is mediated by 2 different enzymes that are spatially separated in the body. The liver-specific TRP 2,3-dioxygenase (TDO) is responsible for the majority of TRP degradation and plays an essential role in the homeostasis of systemic TRP metabolism (31, 32). The extrahepatic indoleamine 2,3 dioxygenases (IDO1 and IDO2) catalyze the same step of TRP catabolism as TDO; however, these enzymes are expressed in a wide number of tissues, most notably the immune system and the gut (33, 34). Under basal conditions, IDO activity is negligible; however, its regulation and activity are dramatically induced by inflammatory cytokines such as TNF-α and IFN-γ and repressed by antiinflammatory cytokines (35).

The induction of IDO activity by inflammatory cytokines is likely responsible for the depletion of TRP and concomitant increase in levels of KYNs seen in older and frail individuals and in the IL-10^tm^ mouse as these all have heightened levels of inflammation. This relationship between inflammatory cytokines and the production of KYNs is demonstrated in this study by the strong correlation observed between the determined KYN levels and the inflammatory cytokine levels. Our findings related to the TRP degradation pathway highlight the potentially important role for this pathway in the decline of multiple systems, which is often observed in frailty.

Several of the defining features of physical frailty — weakness, slowness, and activity — are governed by neuromuscular action, thus making the neuromuscular function an inextricable factor in frailty. The NMJ is the interface between the central nervous system and the musculoskeletal system and is increasingly implicated as a key site where age-related changes contribute to a progressive decline in muscle mass and strength with aging (4). Emerging evidence indicates that degeneration at the NMJ is an early event of age-related muscle weakness (36, 37). At the same time, proximal motor axons of the corresponding NMJs are relatively preserved, suggesting that dying-back axonal degeneration of motor neuron is an underlying pathology of age-associated degeneration of the neuromuscular system (38). Interestingly, dying-back axonal degeneration is a form of neuronal cell death when a neuron is under systemic metabolic stress, and motor neurons in particular are very susceptible to any changes in energy-producing metabolic pathways. In our study, the neuromuscular system of the IL-10^tm^ mouse recapitulated some cardinal features of aging, such as partial denervation at NMJ, reduction in muscle mass, and reduced isometric force. Given that our metabolomic profiling from both mice and humans showed increased activity of the TRP degradation pathway toward the production of neurotoxic KYNs, we hypothesized that increased levels of neurotoxic KYNs mediate neuromuscular defects with aging by causing the metabolic stress in neurons, which leads to dying-back axonal degeneration (schematic summarizing the impact of altered KYNs on different tissues in frailty is shown in Supplemental Figure 3). KYNs have been implicated as etiological factors in several human diseases (18, 39). However, few studies have explored the potential toxicity of KYNs in the peripheral nervous system.

Our measures of in vivo nerve-evoked muscle contractility provide insight into deleterious effect of increased KYNs on neuromuscular integrity. In IL-10^tm^ mice, we observed a decrease in total isometric force that was largely accounted for by a loss in muscle mass and further showed a depressed maximal rate of contraction. Together, these deficits align with decreased function and loss of power in the aging human, and both are consistent with chronic neurogenic atrophy as the inciting cause.

In this study, we saw increased partial denervation in older IL-10^tm^ mice compared with controls — a result that aligns with our previous work showing distal axon degeneration in the aging peripheral motor nerve system (38). A recent study in humans has reported increases in markers of denervation in the vastus lateralis muscle of frail elderly females compared with those of young inactive females (40). Therefore, we propose that the motor nerve degeneration is responsible for the muscle and myofiber atrophy seen in this study. While direct myotoxicity of KYNs is possible, it has not been reported. We will address this possibility in future studies. Furthermore, studies detailing the temporal changes in isometric force, muscle mass, and motor-neuron electrophysiology may reveal a complex trajectory of mechanisms by which KYNs interact with chronic inflammation to predispose neuromuscular decline in aging.

In the syndrome of frailty, multiple systems decline in parallel, leaving the frail, older adult more vulnerable to a host of adverse health outcomes (28, 41, 42). Chronic inflammatory pathway activation has long been thought to influence some of these declines, although exact mechanisms are not always clear. The pleiotropic nature of the biology of KYNs hints at the potential for its impact on multiple tissues and physiological systems, thus providing a direct link between chronic inflammation and multisystem decline.

Our observation of altered levels of KYNs may have important implications on brain function and physiology in older and frail individuals. 3HK and QA are toxic intermediates of the KYN pathway. QA is an excitotoxin (43), and 3HK is cytotoxic and causes oxidative stress in vitro (44). While QA cannot cross the blood-brain barrier, KYN and 3HK can cross the blood-brain barrier and can subsequently be synthesized into QA (45). Thus, blood levels of KYNs can directly influence brain levels of neurotoxic KYNs. These findings highlight a potential mechanistic linkage between mild cognitive impairment (46) and chronic inflammation and frailty. Our findings are also in agreement with the 5HT-KYN hypothesis of depression (47–49), which proposes that depression can result from an inflammation-driven shunting of TRP toward KYN production and away from 5HT production. This may explain the high prevalence of depression among frail subjects (50, 51).

Prior research suggests that increased KYNs play a role in the development of chronic renal failure and shows that the severity of kidney disease is proportional to the accumulation of KYNs (52). Our data are in agreement with this prior research implicating kidney function impairment and the buildup of toxic metabolites. Several metabolite measurements were indicative of decreased kidney and endothelial function in the older and frail subjects. The GABR (arginine/[citrulline+ ornithine]) was significantly lower in older subjects compared with young. This ratio gives an approximation of the bioavailability of arginine and nitric oxide (NO) production and has been associated with increased risk of cardiovascular disease (53). Free ADMA competes for the active site of NOS and may account for reduced NO generation in some disease states, causing oxidative stress and endothelial dysfunction (54, 55). Citrulline is also linked to L-arginine metabolism; in chronic kidney disease subjects, renal citrulline uptake is diminished, the amount of citrulline converted to arginine in the kidney is reduced, and plasma citrulline levels and turnover are elevated (56). Older subjects had increased SDMA, ADMA, and citrulline, as well as increased ratios of SDMA/arginine, total dimethylarginine/arginine, and citrulline/arginine (Table 2). Together, these results indicate that frail and older subjects have decreased kidney function relative to young adults. Under normal circumstances, the majority of KYN is excreted in the urine (57); however, poor kidney function could lead to increased levels of circulating KYNs. Whether the observed increase in KYNs in old and frail subjects is a result of overproduction or underexcretion is still unclear. Further research is necessary to understand the factors that contribute to the accumulation of these metabolites in older frail adults.

Previous studies have corroborated our findings of increased TRP degradation and an increased KYN/TRP ratio with aging and frailty (58, 59). Our study provides additional evidence from frail mice and humans, as well as a thorough, detailed pathway analysis measuring several additional bioactive metabolites from the TRP degradation pathway. This analysis led to the observation of increased levels of toxic KYNs with age and of further increases in deleterious KYNs with frailty. Our study has also revealed the association between 3HK and walking speed.

There are limitations to this study. While we were able to show a strong association between alterations in KYN pathway metabolites, physical frailty, neuromuscular decline, and chronic inflammation in an animal model and in human subjects, due to the cross-sectional design of these studies and modest sample size, the causal relations between them have yet to be fully established. In future studies, we will longitudinally explore the mechanisms of KYN neurotoxicity and rescue the phenotypes of neuromuscular degeneration and frailty via pharmacological or genetic manipulation of the KYN pathway. Eventually, clinical trials will be needed to determine whether manipulation of the KYN pathway may lead to the prevention of neuromuscular decline and physical frailty with aging, as well as reducing overall morbidity and mortality in late life.

In conclusion, we identified major, inflammation-driven derangement in the TRP degradation pathway with the accumulation of cyto- and neurotoxic metabolites in aging and frailty. Furthermore, given the apparent linkages to neuromuscular integrity seen here, our findings highlight a potentially important role for this pathway in functional decline and serve as a vista for future studies focusing on the interface between chronic inflammation and the development of multisystem decline often observed in frailty.

## Methods

### Mouse experiments.

Comparisons between young (5 months old), middle-aged (12 months old), and old (22 months old) female IL-10 deficient B6.129P2-IL 10^tm1Cgn^/J (IL-10^tm^) mice and sex-matched C57BL/6J (control) mice were undertaken. IL-10^tm^ mice were homozygous for the IL-10^tm1Cgn^ targeted mutation backcrossed on the B6 background. All IL-10^tm^ mice were bred in the Johns Hopkins University Bayview Vivarium and maintained under SPF barrier conditions. Female C57BL/6J controls were bred at the Johns Hopkins University Bayview Vivarium or for metabolomic experiments, purchased from The Jackson Laboratory. Mice purchased from The Jackson Laboratory were housed at Johns Hopkins University Bayview Vivarium for a minimum of 2 weeks before any experiment. All mice were housed under the same conditions and fed the same chow at the Johns Hopkins University Bayview Vivarium or at the National Institute on Aging Extramural Branch in Baltimore for all experiments in the study.

Mice were housed in 75 in^2^, autoclave sterilized, high-temperature polycarbonate shoebox cages in ventilated racks (Allentown Inc.) containing autoclaved corncob bedding (Harlan Teklad), autoclaved mouse chow 2018SX (Harlan, Teklad), and reverse osmosis–filtered hyperhlorinated water dispensed through an in-cage automatic watering system (Edstrom Industries). Rooms were maintained at 22°C ± 3.6°C on a 14-hour light/10-hour dark cycle with automated monitoring by Siemens Building Technologies Inc. Cages were changed every 2 weeks in laminar airflow change stations (The Baker Co.) with surface cleaning and disinfection with MB-10 disinfectant (Quip Laboratories Inc.). All caging was sanitized by automatic cage washing systems and autoclaved before use.

### Plasma metabolomic methods.

Metabolites were extracted, and concentrations were obtained using the AbsoluteIDQ kit p180 (Biocrates Life Science AG) following the manufacturers protocol for the API5500 LC/MS/MS System (AB SCIEX) running with Analyst 1.5.2 software equipped with an electrospray ionization source, a Shimadzu CBM-20A command module, a LC-20AB pump, a Shimadzu SIL-20AC-HT autosampler, and a CTO-10Ac column oven heater (60). Briefly, 10 μL of plasma-EDTA samples were pipetted onto the center of the spots in each well of a 96-well Biocrates kit. The samples were dried with a Microvap 118 from Organomation Associate nitrogen evaporator at room temperature (RT) for 30 minutes. A total of 50 μL of 5% PITC reagent was added and incubated for 20 minutes, and the plate was dried under nitrogen for 1 hour. A total of 300 μL of 5 mM ammonium acetate in methanol was added to each well and incubated at RT on a shaker (450 rpm) for 30 minutes. The plate was then centrifuged at 100 *g* for 2 minutes, resulting in about 350 μL of sample extracts in the capture plate; 50 μL of each sample was transferred to the empty 96–deep well plate; and 10 μL of each sample was transferred from the capture plate to the empty 96–deep well plate labeled as “Use for LC.” The extracts were diluted for LC by adding 450 μL of 40% methanol (in HPLC-grade water) to each well. The extracts were diluted for flow injection analysis (FIA) by adding 490 μL of FIA running solvent (Biocrates solvent diluted with HPLC grade methanol). The liquid chromatography–mass spectrometry (LC-MS) plate was run first, with 10 μL injected onto the Eclipse XDB C18, 3.5 μm, and 3.0 × 100 mm with a Phenomenex C18 Security Guard Cartridge, 3.0 mm internal diameters (ID). The mobile phase consisted of solvent A (water containing 0.2% formic acid) and solvent B (acetonitrile containing 0.2% formic acid), with the following gradient: 0–0.5 minutes 0% B, 5.5 minutes 95% B; 6.5 minutes 95% B; 7.0 minutes 0% B; 9.5 minutes 0% B. Evaluation of the samples was carried out using the MetIDQ software. The FIA plate was run with 20 μL injection directly into the MS at a flow of 30 μL/min with water/acetonitrile (1:1) containing 0.2% formic acid as the mobile phase, with the following flow rate program: 0–1.6 minutes 30 μL/min; 2.4 minutes 200 μL/min; 2.8 minutes 200 μL/min; and 3.00 minutes 30 μL/min. Concentrations were calculated using the Analyst/MetIDQ software. PITC, ammonium acetate, water, methanol, and acetonitrile (LC-MS grade) were purchased from MilliporeSigma. QA measurements are relative and represented by the AUC. Of the 186 preconfigured metabolites and ratios in the kit, 121 metabolites were quantifiable in our serum samples. Metabolites were abbreviated to conserve space, and their full names can be found in the Supplemental Table 8.

### In vivo muscle strength testing.

Nerve-evoked contractile function of gastrocnemius muscles in vivo was evaluated as previously described (61, 62). In brief, anesthetized mice (isoflurane) were placed supine on a warming pad (37°C) of an Aurora 1300A system with knee position fixed and foot secured to the foot-plate of the 300C-FP. Percutaneous nerve stimulation was with brief (100 microsecond) pulses with current adjusted to achieve maximal isometric force. The force versus frequency relationship was determined with 250 ms trains of pulses between 1 and 150 Hz. Data were analyzed with DMA-HT analysis software (Aurora Scientific), evaluated for statistical difference using comparison using SigmaStat 4.0 (Jandel Scientific), and plotted using Origin 2019 (OriginLab).

### Gastrocnemius histology.

Muscle fiber cross-sectional area was determined as described (63). The gastrocnemius muscle from the unstimulated leg was snap-frozen in cryomatrix by submersion in isopentane cooled on dry ice in. Cryosections were taken from the muscle midbelly, air dried to a glass slide, fixed with 4% paraformaldehyde, and labeled with Alexa-647–conjugated wheat germ agglutinin (WGA; 1 mg/mL at 1:5000 in PBS, Thermo Fisher Scientific, W32466) to decorate the extracellular matrix for denoting muscle fiber boundaries. Whole muscle sections were imaged under widefield fluorescence (Nikon Ti2, 20×) and cross-sectional areas of each muscle fiber determined with custom routines in Nikon Elements General Analysis 3 software.

### Immunofluorescent quantification of NMJs.

Extensor digitorum longus (EDL) muscles were dissected, pinned in mild stretch, and fixed by immersion for 20 minutes in 4% paraformaldehyde with PBS (pH 7.4). After rinsing in PBS, muscles were soaked in 15% sucrose with PBS (overnight at 4°C), followed by 30% sucrose with PBS for at least 24 hours. Muscle tissues were embedded in OCT and sectioned with a cryostat (HM 550; Microm GmbH) into 40-mm sections and placed on glass slides for staining. For a presynaptic marker, sections were incubated with *β*III-tubulin (1:500; Promega, catalog G7121) antibody overnight at 4°C, followed by secondary fluorescein isothiocyanate–labeled goat anti-mouse (1:100; Jackson ImmunoResearch, catalog 115-095-205) antibody. Rhodamine bungarotoxin (1:40, Thermo Fisher Scientific,, catalog B13423) was used as a postsynaptic marker. Stained sections were examined under fluorescence and confocal microscopy. The Zeiss LSM Image Examiner version 1.0.0.241 (Carl Zeiss) was used for imaging analysis. We quantitated pre- over postsynaptic NMJ areas, and this method has shown to be effective in detecting differences in NMJ connectivity in published studies (64).

### Murine motor neuron–neuroblastoma cell line cultures and toxicity assay.

Murine motor neuron–neuroblastoma cell line (MN1) was used for the cell model for motor neuron. MN1 is a cholinergic motor neuron cell line derived from a fusion of neuroblastoma cell line (N18TG2) with embryonic mouse spinal cord motor neurons from C57BL/6J mouse. MN1 cell line was provided by Charlotte Sumner (Johns Hopkins University). The cells were grown in plastic tissue culture flasks in complete growth media, according to published protocols (65). At 80% confluence, the cells were transferred into 24-well dishes on coverslips and induced to differentiate in the presence of sodium butyrate (1 mm) and aphidicolin (0.4 mg/mL; both from MilliporeSigma) with regular media. Cells were fed daily with the same media for 2 days before treatment with 3HK or QA at various concentrations. ATP levels of neurons were measured for neurotoxicity in 96-plate format, using ViaLight Plus kit (Lonza, catalog LT07-121).

### Human study design and participants.

One hundred and sixty-six community-dwelling adults, age 20–93 years, living in the Baltimore, Maryland, USA, area were recruited with the goal of developing a discovery set with a wide age range. In order to minimize the pharmacological impact on the metabolomic measurements related to inflammatory pathway activation, exclusion criteria included angiotensin receptor blockers, angiotensin-converting enzymes inhibitors, estrogen replacement therapy, corticosteroids, methotrexate, and nonsteroidal antiinflammatory drugs or other immune-modulating agents. Based on the age distribution, subjects were divided into 2 groups: young adult (age 20–30 years, *n* = 50) and older adult (>70 years, *n* = 116). A uniform structured clinical evaluation was performed on each older adult participant to identify frail individuals. Frailty status was assessed using a commonly used and well-validated frailty phenotype screening tool, which consists of grip strength and walking speed measurements, as well as weight loss, fatigue, and physical activity questions (28).

Data collected on subjects from the population included demographics (age, sex, ethnicity), physiologic (BMI, blood pressure, comorbid conditions, medications), functional (grip strength and walking speed), and outcome (falls and mortality) measures. Study subjects were enrolled with informed consent, and the study protocols had appropriate approval by the Johns Hopkins Medical IRB.

### Serum collection and cytokine measurement procedures.

Blood collection visits were scheduled in the morning. Blood was drawn from each individual participant into serum-separator tubes, which were inverted 5 times, allowed to clot for 30 minutes, and centrifuged at 1,100 *g* for 15 minutes in a fixed angle rotor (4°C). Serum aliquots were then transferred to cryovials and stored at –80°C until the immunoassays were performed. The number of freeze-thaw cycles was limited to ≤ 3. Serum cytokines (IL-6, TNF-α, TNF-αR1, IL-1β) were assayed using a quantitative sandwich ELISA (Mesoscale Diagnostics) following the manufacturer’s protocol. Performance characteristics of the cytokine immunoassay included a lower limit of detection (LLOD) of 0.09 pg/mL and a lower limit of quantitation (LLOQ) of 0.30 pg/mL. Due to sample volume limitations, some metabolite measurements were not available for 3 frail, 12 nonfrail, and 5 young subjects.

### Metabolite measurements.

Metabolites were extracted and concentrations were obtained using the AbsoluteIDQ kit p180 (Biocrates Life Science AG) as detailed above.

### KYN pathway analysis.

Concentrated stock solutions of standards were prepared at 1000 μg/mL and stored at –20^o^C. TRP, PA, and 5HT were dissolved in 50:50 methanol/water with 0.1% formic acid. KYN and 3HK were dissolved in 0.1% formic acid in methanol. XA and KA were dissolved in DMSO. Anthranilic acid (AA) was dissolved in water. The deuterated internal standards were dissolved in the same solvent as their corresponding standard.

Separation of the KYNs was accomplished following a previously published protocol (66). Briefly, a linear gradient was run for 30 minutes at a flow rate of 0.3 mL/min: 0–1 minutes 5% B, 3 minutes 23% B, 3.1–5 minutes 70% B, 5.5–20 minutes 90% B, 20.1 minutes 10% B, and 21 minutes 5% B at 40°C, on an X-Select HSS C18 column (2.1 × 150 mm, 2.5 μm, Waters), with mobile phase A consisting of 0.2% aqueous formic acid and mobile phase B consisting of 0.2% formic acid in methanol. Calibration curves were prepared in 0.1% formic acid in 10:90 methanol/water by a 0.5 serial dilution of standards from 100,000 ng/mL to 195.31 ng/mL for TRP; 5000 ng/mL to 9.77 ng/mL for KYN; 2,500 ng/mL to 4.89 ng/mL for 3HK; 1250 ng/mL to 2.44 ng/mL for 5HT; 1250 ng/mL to 2.44 ng/mL for PA; and 312.50 ng/mL to 0.61 ng/mL for XA, KA, and AA in standard solution. Measurements of KYN and TRP, as well as 5HT, was highly correlated to the results obtained by the Biocrates Kit.

Relative concentrations (μM) of the metabolites were determined in standard solution using area ratios calculated using their corresponding deuterated standard (D_5_-TRP, D_4_-KYN, D_4_-XA, D_5_-KA, D_4_-PA, D_3_-5HT) with the exception of 3HK and AA, where D_4_-KYN was used as their internal standard, and QA, where only the AUC was used and D_4_-PA as the internal standard. As the calibration curve is not carried out in matrix, the reported concentrations in the developed method provide a measure of relative abundance and not absolute quantification, as matrix effects were not considered. A pooled sample of the study subjects was run each day, to which all data were normalized. If significant matrix effects were observed in a sample, that sample was not included in the data analysis. In the sample subset, 3HK levels were near the lowest calibration point. AA had > 20% of the samples that were not detectable across all groups; as a result, it was not included in the data figures.

To 40 μL plasma, 10 μL internal standard, and 10 μL 0.1% formic acid in water was added. Solid-phase extraction cartridges (Oasis HLB, Waters Corp.) were conditioned with 1 mL methanol and then 1 mL water. The samples were added and washed with 100 μL water. Finally, the metabolites were eluted with 1 mL 0.1% formic acid in 95:5 methanol/water and stream dried under nitrogen. The samples were reconstituted in 100 μL 0.1% formic acid in 10:90 methanol/water and transferred to autosampler vials for analysis.

Data were acquired using a Nexera XR HPLC (Shimadzu) coupled with a QTRAP 6500+ (SCIEX) and were analyzed with Analyst 1.6 (SCIEX). The positive ion mode data was obtained using multiple reaction monitoring (MRM). The instrumental source setting for curtain gas, ion spray voltage, temperature, ion source gas 1, and ion source gas 2 were 30 psi, 5500 V, 500°C, 40 psi, and 50 psi, respectively. The collision activated dissociation was set to medium, and the entrance potential was 10 V. The standards (Supplemental Table 9) and internal standards (Supplemental Table 10) were characterized using the MRM ion transitions, declustering potentials (DP), collision energies (CE), and collision cell exit potentials (CXP) listed in Supplemental Tables 9 and 10.

### Statistics.

Comparisons of individual metabolite values between IL-10^tm^ mice and control mice were carried out by 2-sample, 2 tailed *t* tests without assuming consistent SD, with statistical significance determined using the Holm-Bonferroni’s method for multiple comparisons correction with maximum family-wise error rate at 0.05. A *P* value less than 0.05 was considered significant. The association between isometric force versus varying levels of stimulation frequency of the gastrocnemius muscle in vivo was compared between the IL-10^tm^ mice and the control mice using 2-way repeated-measures ANOVA. Between-group differences in body weight, gastrocnemius muscle weight, and kinetics of muscle contraction were tested using 2-sample, 2-tailed *t* tests. A *P* value less than 0.05 was considered significant. Morphological changes in the muscle fiber cross-sectional area were with nonparametric Mann-Whitney *U* test and NMJ were analyzed using 2 tailed *t* test and frequency histogram.

Using human subjects, comparisons of individual metabolite values between young and old subjects were carried out by 2-tailed *t* tests without assuming SD, with statistical significance determined using the Holm-Bonferroni’s method for multiple comparisons correction with maximum family-wise error rate at 0.05. A *P* value less than 0.05 was considered significant. To address skewness in the data, comparisons of distributions of metabolites by age and frailty status (young, nonfrail, frail) were conducted using the Kruskall-Wallace 1-way ANOVA with Dunn’s multiple comparisons test. The ATP levels of MN1 cells under various concentrations of 3HK and QA toxicity levels were analyzed using 1-way ANOVA, followed by Tukey’s multiple comparisons test. Linear regression models were used to estimate the association between metabolite levels and cytokines (IL-6, IL-1, IFN, TNF-α, and TNF-αR1), and functional measures including walking speed and grip strength after adjusting for age. We used logistic regression to estimate the OR of being frail versus nonfrail in relation to blood levels of each metabolite after adjusting for age. In the regression analyses, we log-transformed the cytokine levels to address the skewness in the data distribution of the cytokines, and we standardized the log-transformed cytokine levels and metabolite values to facilitate the comparison of the associations across metabolites. Statistical analyses were performed using GraphPad Prism 6 and SAS version 9.2.

### Study approval.

All animal-related experimental procedures performed in this study were approved by the Johns Hopkins University animal care and use committee (protocol no. MO12M341). All human study subjects were enrolled with informed consent, and the study protocols had appropriate approval by the Johns Hopkins Medical IRB.

## Author contributions

RW, TC, AL, LF, RM, JW, AH, RDC, and PMA participated in study concept and design. PMA was the PI of the study, recruiting the subjects to the study, and was responsible for the acquisition of the functional, demographic, and serum cytokine data. RDC, JW, and RW maintained the aging animal colonies for the mice used in this study. RM and RW were responsible for the acquisition of the metabolomic data. QLX, RW, TC, LF, AL, RM, JW, PMA, and JT participated in the analysis, interpretation of the data, and drafting the manuscript and approved the final version. TC, CW, RW, and AH were responsible for in vivo muscle physiology experiments, staining and analysis of NMJ staining, and in vitro experiments on MN1 motor neuronal cell line. RW, QLX, AL, TC, LF, RM, JW, and PMA were responsible for the critical revision of the manuscript for important intellectual content and approval of the final version. JL was responsible for the acquisition of the metabolomic data. HJ was responsible for in vivo muscle physiology experiments. HY maintained the aging animal colonies for the mice used in this study.MK was responsible for the acquisition of the metabolomic data. RW, TC, JW, RM, QLX, and PMA had full access to all the data in the study and take responsibility for the integrity of the data and the accuracy of the data analysis.

## Supplementary Material

Supplemental data

## Figures and Tables

**Table 1:**
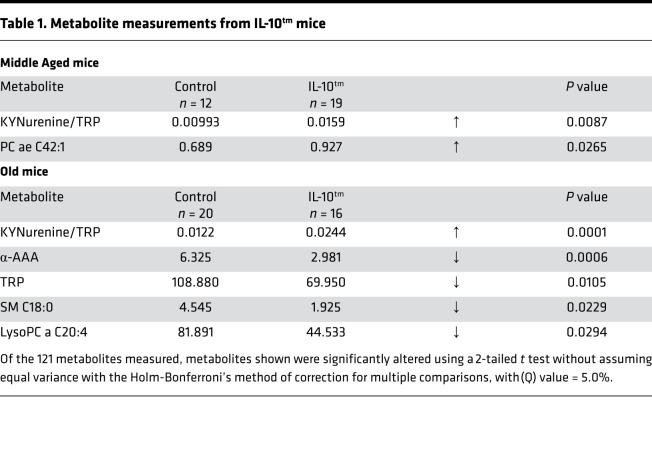
Metabolite measurements from IL-10^tm^ mice

**Table 2:**
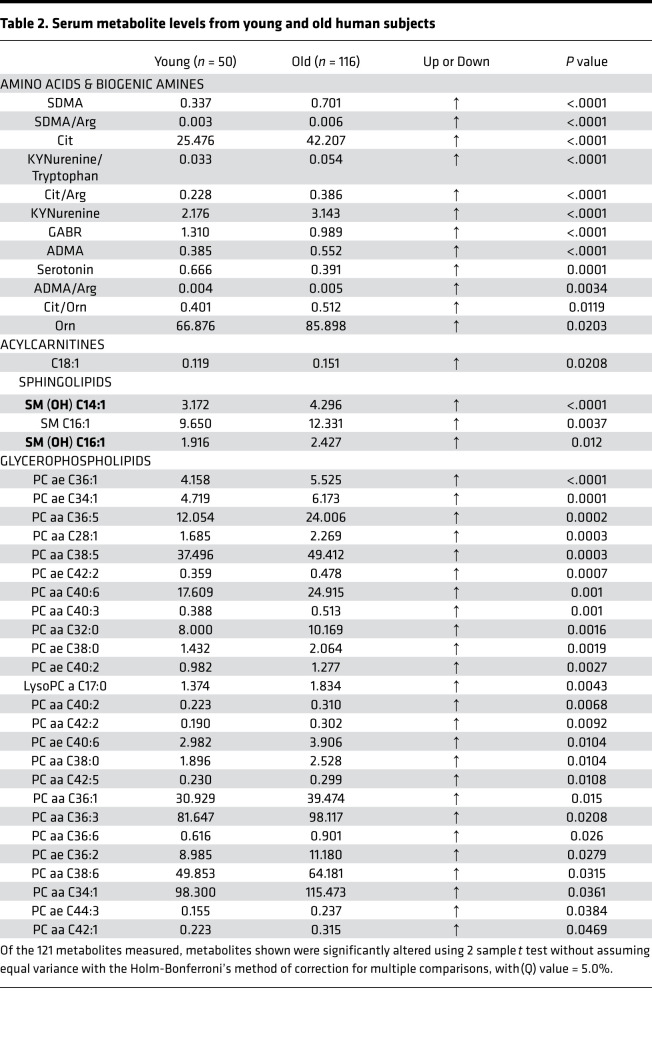
Serum metabolite levels from young and old human subjects

**Table 3:**
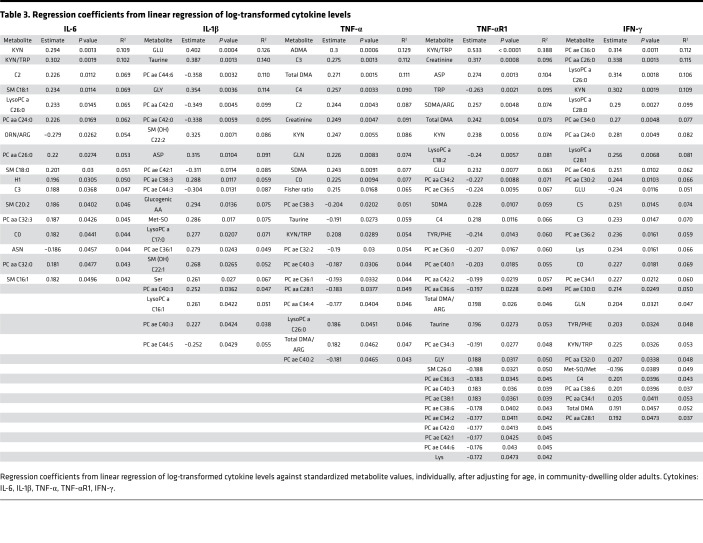
Regression coefficients from linear regression of log-transformed cytokine levels

**Table 4:**
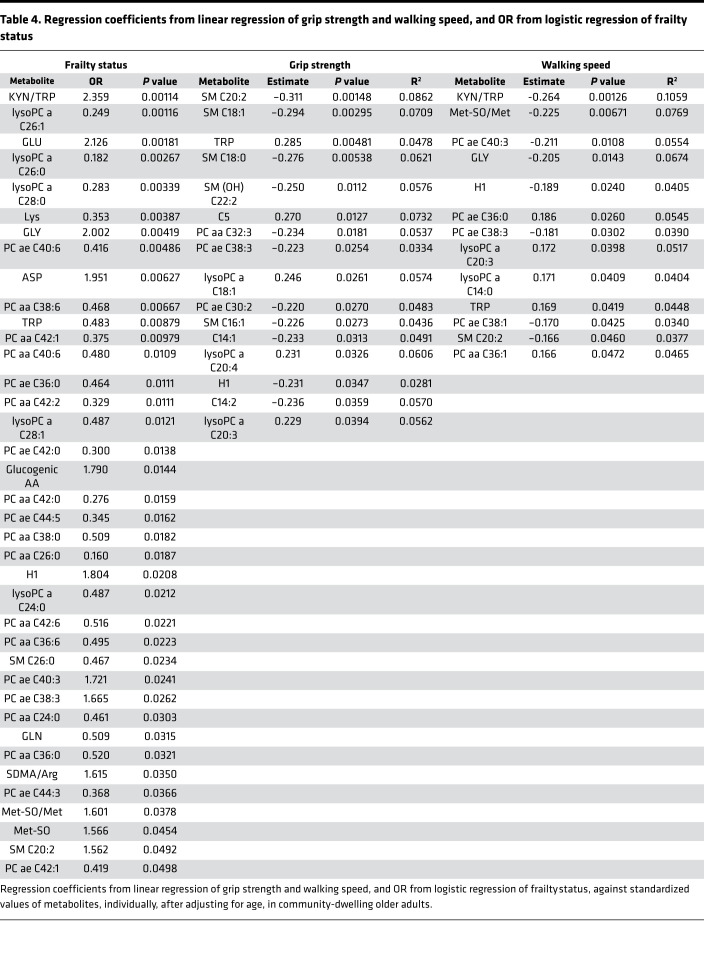
Regression coefficients from linear regression of grip strength and walking speed, and OR from logistic regression of frailty status

## Version 1 08/20/2020

Electronic publication
