# Supplementary material for: Kynurenines link chronic inflammation to functional decline and physical frailty
Source: JCI Insight. 2020 Aug 20;5(16):e136091. doi: 10.1172/jci.insight.136091 (PMC7455140; doi:10.1172/jci.insight.136091)
Supplement: Supplemental data [file jciinsight-5-136091-s263.pdf]

Supplemental Table 1. Young IL 10^{tm} mice metabolite measurements (full table, p-values unadjusted)

Metabolite		Control n=5	IL 10^{tm} n=6		P value
**Cit**		94.45	73.5167	↓	0.0036684
**Orn**		73.55	56.8167	↓	0.0100904
**PC ae C38:2**		4.95467	3.52205	↓	0.0103952
**PC ae C38:3**		1.97635	1.52756	↓	0.0129673
**lysoPC a C14:0**		8.68146	6.66934	↓	0.0206697
**SM (OH) C14:1**		1.72292	1.26118	↓	0.021117
**PC ae C36:3**		2.00604	1.7431	↓	0.0218773
**lysoPC a C16:1**		5.7321	4.32297	↓	0.025716
**PC ae C36:2**		9.64386	8.07114	↓	0.0276785
**SM (OH) C22:2**		1.85736	1.47848	↓	0.032493
**PC aa C36:3**		95.9061	81.4081	↓	0.0334162

Supplemental Table 2. Middle aged IL 10^{tm} metabolite measurements (full table, p-values unadjusted)

Metabolite		Control n=12	IL 10^{tm} n=19		P value
**Kynurenine / Trp**	*	0.0099273	0.0158913	↑	4.44E-05
**PC ae C42:1**	*	0.688724	0.927056	↑	0.0001366
**PC aa C40:6**		36.2821	53.1196	↑	0.0004424
**PC aa C40:4**		1.71318	2.33144	↑	0.0008275
**PC aa C40:1**		0.288054	0.346857	↑	0.0012507
**lysoPC a C26:1**		0.315676	0.434985	↑	0.0012549
**PC ae C42:4**		0.283372	0.385398	↑	0.0013905
**PC ae C40:5**		0.957944	1.27838	↑	0.0016083
**PC ae C36:5**		1.60583	2.41199	↑	0.001617
**PC aa C42:5**		0.226678	0.293754	↑	0.0019018
**PC aa C32:3**		0.177071	0.230479	↑	0.0023928
**SM (OH) C14:1**		1.80553	1.33645	↓	0.0027591
**PC aa C34:3**		10.4825	14.5773	↑	0.0028308
**PC ae C36:4**		2.50391	3.42155	↑	0.0028347
**PC aa C38:4**		116.879	156.955	↑	0.0032746
**Kynurenine**		1.21658	1.63516	↑	0.0035013
**lysoPC a C28:1**		0.636165	0.81616	↑	0.0035348

**PC aa C38:6**	76.1241	106.147	↑	0.0039363
**PC aa C36:5**	6.59467	9.30779	↑	0.0042725
**PC ae C34:3**	0.827801	1.14532	↑	0.0042915
**PC ae C38:5**	2.79781	3.75849	↑	0.0043954
**PC ae C32:1**	0.521774	0.664599	↑	0.0062371
**PC ae C42:0**	1.01761	1.17576	↑	0.0063958
**PC ae C42:5**	0.687499	0.817973	↑	0.0068315
**PC aa C34:4**	0.50192	0.662721	↑	0.0080307
**PC ae C30:2**	0.100309	0.119044	↑	0.0085778
**PC ae C38:0**	2.06255	2.77453	↑	0.0098392
**Serotonin**	9.05709	3.34308	↓	0.0125052
**PC ae C30:1**	0.208129	0.278508	↑	0.0126652
**c4-OH-Pro**	4.46417	5.58789	↑	0.0129227
**PC aa C38:0**	1.22125	1.56823	↑	0.0159307
**PC aa C42:6**	0.468822	0.552634	↑	0.0162391
**PC aa C40:5**	4.05247	5.21375	↑	0.0170496
**PC ae C40:4**	1.64796	2.09069	↑	0.0180223
**PC ae C38:6**	1.43781	1.84396	↑	0.0198495
**PC ae C34:1**	2.34154	2.89283	↑	0.019872
**Gly**	196.983	243.263	↑	0.0202694
**PC aa C38:5**	35.0897	44.7049	↑	0.0204442
**PC ae C32:2**	0.218689	0.26545	↑	0.0214631
**PC aa C42:4**	0.606987	0.665631	↑	0.022113
**PC aa C36:4**	127.586	162.928	↑	0.0225723
**PC aa C36:6**	0.349633	0.451909	↑	0.0270209
**PC aa C42:1**	0.206177	0.246872	↑	0.0301884
**PC ae C38:4**	3.91691	4.87044	↑	0.033847
**PC aa C36:2**	249.656	297.116	↑	0.0373519
**SM C20:2**	0.285843	0.348296	↑	0.0425698
**lysoPC a C18:0**	167.607	199.504	↑	0.0443904
**PC aa C32:1**	3.15613	4.09743	↑	0.0476246
**PC aa C40:3**	0.381395	0.45225	↑	0.047744
**PC ae C36:0**	0.39648	0.476158	↑	0.0478637
**PC aa C38:3**	24.9592	31.1117	↑	0.0492607

Supplemental Table 3. Old age IL 10^{tm} mice metabolite measurements (full table, p-values unadjusted)

ID		Control n=20	IL 10^{tm} n=16		p-value
**Kynurenine / Trp**	*	0.0122069	0.0244054	↑	7.07E-07
**alpha-AAA**	*	6.325	2.98062	↓	2.98E-06
**Trp**	*	108.88	69.95	↓	5.46E-05
**SM C18:0**	*	4.54467	1.92527	↓	0.0001196
**lysoPC a C20:4**	*	81.891	44.5328	↓	0.0001547
**lysoPC a C16:1**	*	4.88079	2.87555	↓	0.0002673
**lysoPC a C18:1**		52.288	34.843	↓	0.0007301
**PC ae C34:2**		1.89739	2.73332	↑	0.0012113
**lysoPC a C16:0**		233.357	163.846	↓	0.0014066
**PC aa C36:0**		2.41212	1.65315	↓	0.0017975
**SM C18:1**		1.19859	0.760069	↓	0.0025144
**lysoPC a C24:0**		0.949746	0.710284	↓	0.0032113
**Fisher ratio**		1.95602	2.34761	↑	0.0034275
**PC aa C40:3**		0.272867	0.351153	↑	0.0037729
**PC ae C42:1**		0.563257	0.879288	↑	0.0038765
**SM (OH) C14:1**		1.35906	0.945649	↓	0.0043295
**lysoPC a C18:2**		164.512	125.249	↓	0.0044795
**PC ae C42:3**		0.895231	0.624062	↓	0.005439
**PC ae C38:3**		0.905353	1.24644	↑	0.0056297
**Kynurenine**		1.2398	1.62625	↑	0.0075272
**C16:2-OH**		0.0707002	0.079037	↑	0.0076157
**PC aa C34:1**		49.5688	36.2751	↓	0.010824
**PC ae C42:0**		0.866178	1.03258	↑	0.0110601
**PC ae C36:2**		4.78344	6.3887	↑	0.0114751
**PC ae C36:3**		1.0802	1.35751	↑	0.0117932
**SM (OH) C16:1**		0.397161	0.29129	↓	0.0125727
**SM (OH) C24:1**		0.473738	0.381408	↓	0.0170087
**Thr**		262.065	173.288	↓	0.0186021
**PC aa C40:6**		57.7794	40.2682	↓	0.0195769
**PC aa C38:6**		92.2034	64.7815	↓	0.0216498
**PC aa C40:2**		0.204337	0.254761	↑	0.0225663
**PC ae C38:2**		2.27925	3.18833	↑	0.0241852
**lysoPC a C18:0**		191.04	144.447	↓	0.0246072
**Phe**		74.175	90.5813	↑	0.0265114
**Serotonin**		4.71245	1.76533	↓	0.0271002

**PC ae C38:0**	1.90518	1.46673	↓	0.0316329
**PC aa C36:3**	44.266	55.8637	↑	0.0326819
**PC aa C32:0**	9.2091	7.48208	↓	0.0410201
**C16:1-OH**	0.156217	0.168695	↑	0.0429336
**PC aa C42:5**	0.205434	0.242672	↑	0.0449734
**PC ae C30:2**	0.0993353	0.0837001	↓	0.0455536
**PC aa C24:0**	0.33076	0.257438	↓	0.0475861
**PC ae C44:5**	0.289386	0.250126	↓	0.0477549
**Tyr / Phe**	0.823069	0.728002	↓	0.0479037

Supplemental Table 4. Demographics of young versus old including age, sex, BMI, systolic BP, Diastolic BP, IL6, TNFα, TNFα R1 (mean & SD).

	Young (n=50)	Old (n=116)	p-value
Age , mean(std)	25.6 (6.2)	77.6 (5.9)	<0.0001
Gender, male, n(%)	34 (68.0)	66 (56.9)	0.18
BMI	26.1 (6.6)	28.0 (5.9)	0.074
Systolic BP	113.9 (14.6)	132.8 (20.1)	<0.0001
Diastolic BP	73.3 (10.9)	73.4 (11.3)	0.96
IL6	0.69 (0.63)	2.04 (4.45)	0.046
TNFa	2.01 (1.16)	3.07 (1.28)	<0.0001
TNFa receptor1	793.65 (298.20)	1454.01 (659.77)	<0.0001

Supplemental Table 5. Average CV's for metabolites in 14 non-frail individuals CV= stdev (of all 4 visits)/ average (of all 4 visits)

Individual variability in blood metabolites can provide information which can help identify candidate metabolites for biomarkers of aging or disease. In order to understand the variability of the metabolites we measured, we performed metabolomics analysis on serum samples collected from fourteen pre-frail individuals four times over a six-month timespan at two-month intervals. This longitudinal collection allowed for the quantification of the variance of each metabolite over the six-month interval. We calculated the average (intra-assay) CV for each metabolite using the CV over the six-month period for each metabolite for each individual patient, and then averaging the CV's from all fourteen subjects. Most of the metabolites had relatively low CV with only 16 metabolites having CV's > 0.3

Metabolite	Average CV	Standard Deviation
PC aa C34:2	0.078	0.04
PC aa C36:2	0.112	0.051
Fisher ratio	0.114	0.044
Non essential AA	0.129	0.061
PC aa C36:4	0.134	0.074
Tyr / Phe	0.134	0.081
PC aa C34:1	0.137	0.064
PC aa C38:4	0.138	0.075
PC ae C42:5	0.14	0.098
Total AA	0.143	0.063
PC ae C44:3	0.148	0.082
PC aa C38:6	0.15	0.096
PC ae C42:1	0.151	0.083
PC ae C44:6	0.151	0.105
Glucogenic AA	0.153	0.118
PC ae C40:4	0.154	0.123
PC ae C34:1	0.155	0.09
PC aa C40:6	0.156	0.091
PC aa C42:5	0.156	0.075
PC ae C40:2	0.156	0.114
PC ae C44:5	0.157	0.09
PC aa C42:0	0.158	0.09
PC aa C36:3	0.158	0.091
PC ae C40:6	0.159	0.11

Phe	0.162	0.093
SM C16:1	0.162	0.11
PC ae C42:4	0.163	0.127
PC aa C40:5	0.163	0.08
PC ae C42:2	0.164	0.089
SM C24:1	0.165	0.105
PC ae C38:4	0.165	0.12
SM C16:0	0.167	0.093
PC aa C28:1	0.167	0.1
PC aa C38:5	0.168	0.097
PC aa C42:6	0.168	0.091
Orn / Arg	0.168	0.079
Gly	0.168	0.116
Val	0.171	0.074
PC ae C40:3	0.171	0.13
PC ae C36:4	0.171	0.104
PC aa C32:0	0.171	0.082
PC aa C42:1	0.172	0.107
PC ae C36:1	0.172	0.133
PC aa C38:3	0.173	0.088
PC aa C42:2	0.173	0.093
PC aa C40:3	0.174	0.08
PC ae C40:1	0.174	0.11
PC ae C40:5	0.175	0.114
SM C26:1	0.175	0.1
Gln	0.175	0.121
SM (OH) C16:1	0.175	0.105
SM (OH) C14:1	0.177	0.107
PC aa C38:0	0.178	0.107
Ala	0.179	0.126
PC ae C38:6	0.18	0.127
PC ae C38:5	0.181	0.114
PC ae C42:3	0.181	0.121
PC ae C38:0	0.183	0.12
PC aa C40:4	0.183	0.093
PC ae C34:2	0.184	0.125
PC ae C34:0	0.186	0.11
PC aa C42:4	0.187	0.064
PC ae C32:2	0.188	0.129
AAA	0.188	0.11
SM C24:0	0.188	0.101
PC ae C36:5	0.189	0.108
Total DMA / Arg	0.189	0.11

SM C18:0	0.189	0.086
SM C18:1	0.189	0.1
C2 / C0	0.189	0.133
C18:2	0.19	0.091
C0	0.19	0.112
PC ae C36:3	0.191	0.126
PC aa C32:3	0.192	0.113
SM (OH) C22:2	0.194	0.112
SM (OH) C24:1	0.194	0.106
PC aa C36:0	0.196	0.105
PC ae C38:3	0.196	0.126
lysoPC a C18:0	0.197	0.101
PC ae C32:1	0.198	0.109
lysoPC a C16:1	0.199	0.126
SM (OH) C22:1	0.199	0.109
PC ae C36:0	0.199	0.113
PC aa C34:3	0.199	0.089
C4	0.2	0.09
PC ae C36:2	0.2	0.174
Kynurenine / Trp	0.201	0.081
PC aa C36:1	0.202	0.107
lysoPC a C16:0	0.202	0.096
PC aa C40:2	0.205	0.112
Met-SO / Met	0.208	0.082
SM C26:0	0.208	0.113
lysoPC a C20:4	0.21	0.087
lysoPC a C17:0	0.21	0.148
Lys	0.211	0.122
PC aa C30:0	0.212	0.083
PC aa C36:6	0.213	0.111
ADMA / Arg	0.213	0.082
PC ae C38:2	0.214	0.115
Pro	0.214	0.071
Essential AA	0.216	0.115
Arg	0.218	0.117
PC ae C34:3	0.219	0.143
Cit / Arg	0.22	0.105
lysoPC a C18:1	0.221	0.117
SM C20:2	0.221	0.108
PC ae C30:2	0.223	0.101
PC aa C34:4	0.223	0.105
Trp	0.224	0.158

total DMA	0.226	0.142
Thr	0.226	0.09
PC ae C44:4	0.226	0.155
Ser	0.227	0.089
C18:1	0.227	0.126
Tyr	0.231	0.12
H1	0.231	0.139
Cit	0.234	0.092
PC aa C36:5	0.235	0.116
Orn	0.236	0.137
C2	0.238	0.131
PC aa C32:1	0.239	0.096
Cit / Orn	0.241	0.099
lysoPC a C20:3	0.241	0.081
PC aa C32:2	0.242	0.086
Kynurenine	0.247	0.152
SDMA / Arg	0.249	0.178
BCAA	0.253	0.127
C3	0.254	0.124
Glu	0.262	0.081
Asn	0.262	0.126
ADMA	0.267	0.176
PC aa C38:1	0.272	0.164
SDMA	0.28	0.233
Taurine	0.28	0.17
Serotonin / Trp	0.297	0.183
C5	0.301	0.215
Putrescine / Orn	0.302	0.194
lysoPC a C18:2	0.303	0.134
Serotonin	0.309	0.108
Met	0.31	0.176
PC ae C38:1	0.321	0.159
SM C22:3	0.326	0.308
Ile	0.33	0.168
Leu	0.331	0.169
Met-SO	0.349	0.211
Asp	0.385	0.15

Supplemental Table 6. Demographics of all older patients stratified by risk status into non-frail and frail groups

	Non-frail (n=83)	Frail (n=33)	p-value
Age , mean(std)	76.7 (5.7)	79.8 (5.9)	0.01
Gender, male, n(%)	39 (47.0)	27 (81.2)	0.0008
BMI	26.65 (3.45)	31.4 (8.5)	<0.0001
Systolic BP	133.03 (17.17)	132.4 (25.0)	0.066
Diastolic BP	74.19 (11.27)	71.5 (11.5)	0.53
IL6	1.96 (3.69)	2.22 (1.76)	0.22
TNFa	3.07 (1.35)	2.73 (0.93)	0.058
TNFa receptor1	1288.01 (580.40)	1862.70 (677.35)	0.0002
Walking time for 4m (sec)	3.75 (0.93)	7.77 (3.58)	<0.0001
Grip strength	26.65 (9.93)	19.04 (6.57)	<0.0001

Supplemental Table 7. Downstream kynurenine pathway metabolites & ratios ranked correlations with IL-6, TNFα, TNFαR1, and IFNγ levels, and frailty status, walking speed, and grip strength

IL-6

Metabolite	Estimate	P-value	R^2
KYN/PA	0.276	0.00995	0.109

TNFα

Metabolite	Estimate	P-value	R^2
TRP/5HT	3.324	0.00229	0.10390
KYN/5HT	2.633	0.00281	0.07430
AA/5HT	0.923	0.0192	0.0759
3HK/5HT	0.532	0.0315	0.0433
XA/KA	-0.253	0.0323	0.0719
5HT/KA	-0.232	0.0364	0.0769
TRP/KA	-0.220	0.0374	0.0607
KYN/QA	0.236	0.0387	0.0649

TNFαR1

Metabolite	Estimate	P-value	R^2
KYN/PA	0.360	0.000776	0.1574
KYN/QA	0.338	0.00110	0.1546
KA/XA	0.350	0.00112	0.158
TRP/3HK	-0.330	0.00113	0.1303
XA/TRP	0.298	0.00370	0.1185
KA/QA	0.295	0.00442	0.1155
KA	0.295	0.00580	0.1119
KA/PA	0.281	0.00730	0.1089
TRP/AA	-0.306	0.0147	0.126
TRP/KA	-0.236	0.0252	0.0904
3HK/PA	0.230	0.0293	0.0621
XA/KYN	-0.218	0.0383	0.0603

IFNγ

Metabolite	Estimate	P-value	R^2
KA/KYN	-0.286	0.0101	0.108
KYN/AA	0.289	0.0210	0.119

Frailty Status

Metabolite	OR	P-value
3HK/TRP	2.683	0.00251
TRP/AA	0.281	0.0179
KA/PA	2.043	0.0314
PA/3HK	0.377	0.0321
KYN/3HK	0.418	0.0343
3HK/XA	1.797	0.0371
XA/AA	0.342	0.0374
KA/KYN	1.845	0.0386
3HK	1.786	0.0393
KA/XA	1.928	0.0415

Grip Strength

Metabolite	Estimate	P-value	R^2
TRP/QA	0.197	0.0211	0.1406
TRP/3HK	0.187	0.0350	0.1473
TRP/XA	0.189	0.0405	0.1189

Walking Speed

Metabolite	Estimate	P-value	R^2
TRP/XA	0.308	0.00148	0.14020
3HK/TRP	-0.308	0.00158	0.14580
3HK/PA	-0.243	0.0154	0.1096
TRP/KA	0.231	0.0179	0.0930
5HT/TRP	-0.199	0.0355	0.0424
3HK	-0.205	0.0401	0.0720
PA/KA	0.201	0.0420	0.0938

Supplemental Table 8. Metabolite abbreviation list

**Acylcarnitines**			
C0	Carnitine	C10:1	Decenoylcarnitine
C2	Acetylcarnitine	C10:2	Decadienylcarnitine
C3	Propionylcarnitine	C12	Dodecanoylcarnitine
C3:1	Propenoylcarnitine	C12:1	Dodecenoylcarnitine
C3-OH	Hydroxypropionylcarnitine	C12-DC	Dodecanedioylcarnitine
C4	Butyrylcarnitine	C14	Tetradecanoylcarnitine
C4:1	Butenylcarnitine	C14:1	Tetradecenoylcarnitine
C4-OH(C3-DC)	Hydroxybutyrylcarnitine	C14:1-OH	Hydroxytetradecenoylcarnitine
C5	Valerylcarnitine	C14:2	Tetradecadienylcarnitine
C5:1	Tiglylcarnitine	C14:2-OH	Hydroxytetradecadienylcarnitine
C5:1-DC	Glutaconylcarnitine	C16	Hexadecanoylcarnitine
C5-DC(C6-OH)	Glutarylcarnitine (Hydroxyhexanoylcarnitine)	C16:1	Hexadecenoylcarnitine

C5-M-DC	Methylglutarylcarnitine	C16:1-OH	Hydroxyhexadecenoylcarnitine
C5-OH (C3-DC-M)	Hydroxyvalerylcarnitine (Methylmalonylcarnitine)	C16:2	Hexadecadienylcarnitine
C6 (C4:1-DC)	Hexanoylcarnitine (Fumarylcarnitine)	C16:2-OH	Hydroxyhexadecadienylcarnitine
C6:1	Hexenoylcarnitine	C16-OH	Hydroxyhexadecanoylcarnitine
C7-DC	Pimelylcarnitine	C18	Octadecanoylcarnitine
C8	Octanoylcarnitine	C18:1	Octadecenoylcarnitine
C9	Nonaylcarnitine	C18:1-OH	Hydroxyoctadecenoylcarnitine
C10	Decanoylcarnitine	C18:2	Octadecadienylcarnitine
**Amino Acids & Biogenic Amines**			
Ala	Alanine	Ac-Orn	Acetylornithine
Arg	Arginine	ADMA	Asymmetricdimethylarginine
Asn	Asparagine	SDMA	Symmetricdimethylarginine
Asp	Aspartate	total DMA	Total dimethylarginine
Cit	Citrulline	alpha-AAA	alpha-Aminoadipic acid
Gln	Glutamine	Carnosine	Carnosine
Glu	Glutamate	Creatinine	Creatinine
Gly	Glycine	Histamine	Histamine
His	Histidine	Kynurenine	Kynurenine
Ile	Isoleucine	Met-SO	Methioninesulfoxide
Leu	Leucine	Nitro-Tyr	Nitrotyrosine
Lys	Lysine	OH-Pro	Hydroxyproline
Met	Methionine	PEA	Phenylethylamine
Orn	Ornithine	Putrescine	Putrescine
Phe	Phenylalanine	Sarcosine	Sarcosine
Pro	Proline	Serotonin	Serotonin
Ser	Serine	Spermidine	Spermidine
Thr	Threonine	Spermine	Spermine
Trp	Tryptophan	Taurine	Taurine
Tyr	Tyrosine		
Val	Valine		
**Monosaccharides**			
H1	Sum of Hexoses (including Glucose)		

SM sphingomyelin

PC Phosphatidylcholine

Aa acyl-acyl

Ae acyl-alkyl

Supplemental Tables 9 & 10. The standards (**Supplemental Table 9**) and internal standards (**Supplemental Table 10**) were characterized using the MRM ion transitions and their voltages: declustering potentials (DP), collision energies (CE), and collision cell exit potentials (CXP) are listed below. Quinolinic Acid was characterized using spectra for picolinic acid and is reported as only the area under the curve ratio with the internal standard.

Supplemental Table 9.

**Analyte**	**Q1/Q3**	**DP**	**CE**	**CXP**
3-Hydroxy Kynurenine	226.0/209.1	20	11	10
Kynurenine	209.0/94.0	20	19	15
Xanthurenic Acid	206.2/160.0	56	27	10
Kynurenic Acid	189.9/144.0	55	25	8
Picolinic Acid	124.0/78.0	10	25	10
Tryptophan	205.1/118.0	39	35	11
Anthranilic Acid	138.2/120.0	36	15	8
Serotonin	177.0/160.1	26	13	8

*Quinolinic Acid was characterized using spectra for picolinic acid and is reported as only the area under the curve ratio with the internal standard.

Supplemental Table 10.

**Internal Standard**	**Q1/Q3**	**DP**	**CE**	**CXP**
D_4-KYN	231.0/98.0	20	19	15
D_4-XA	210.0/164.0	56	27	10
D_5-KA	195.0/149.2	55	27	8
D_4-PA	128.1/82.1	10	25	10
D_5-TRP	210.1/122.0	30	35	10
D_3-5HT	181.1/164.1	26	13	10

Supplemental Figure 1

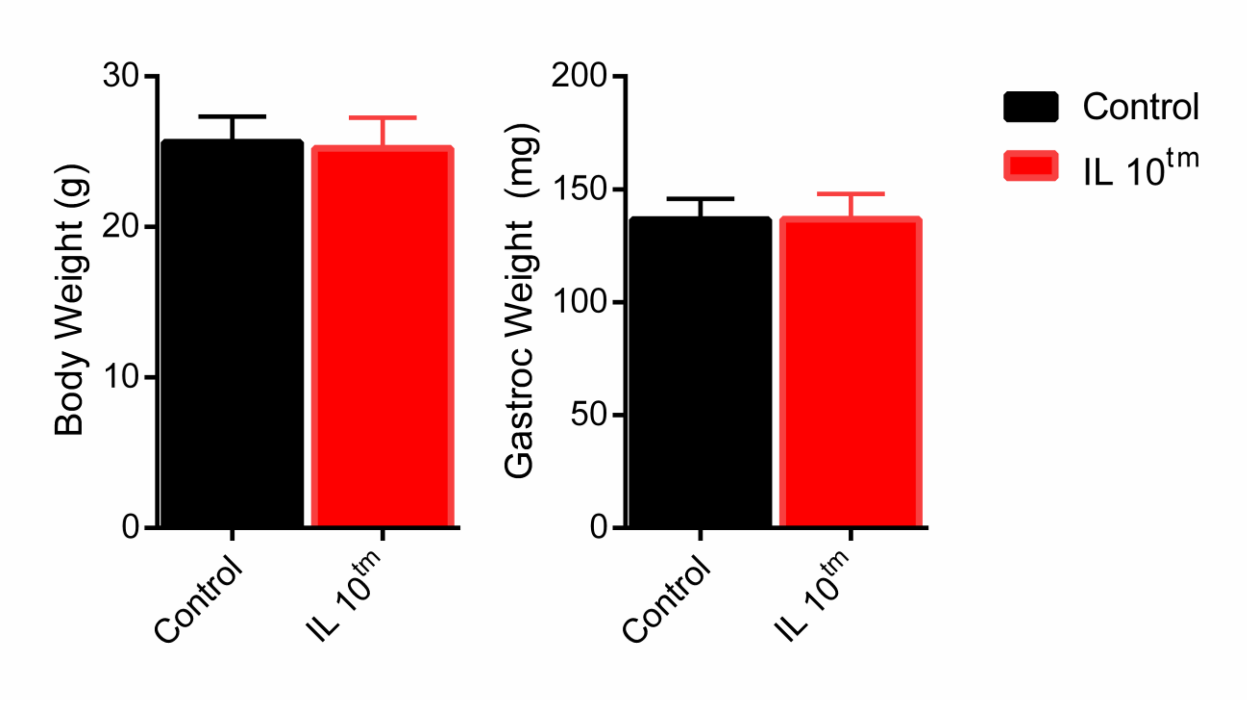

Supplemental Figure 1. Body weight and gastrocnemius weight from 3 month old IL 10^{tm} and control mice (n=10 per group)

Supplemental Figure 2

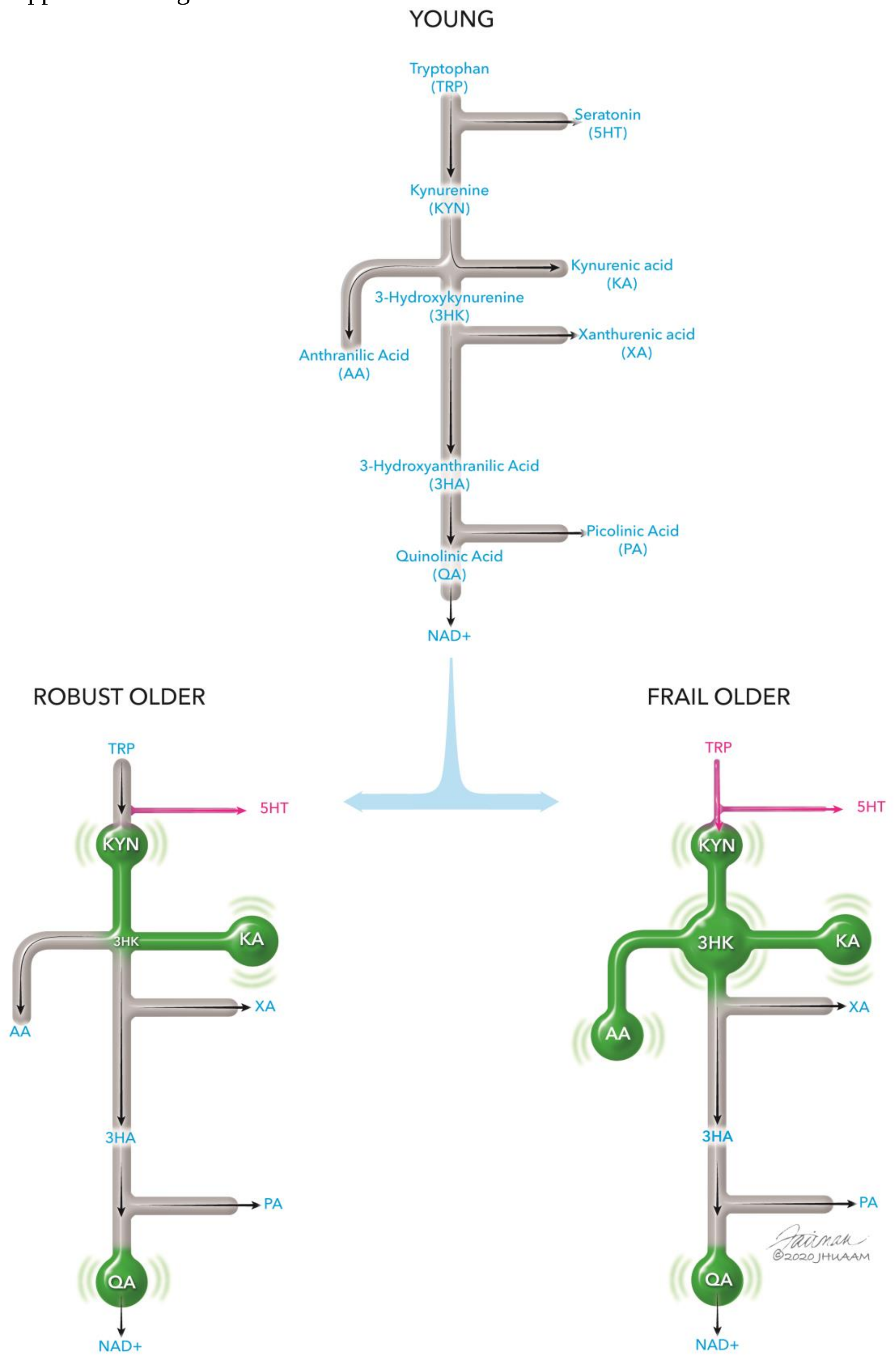

Supplemental Figure 2. Summary of kynurenine pathway changes with age & frailty

Supplemental Figure 3

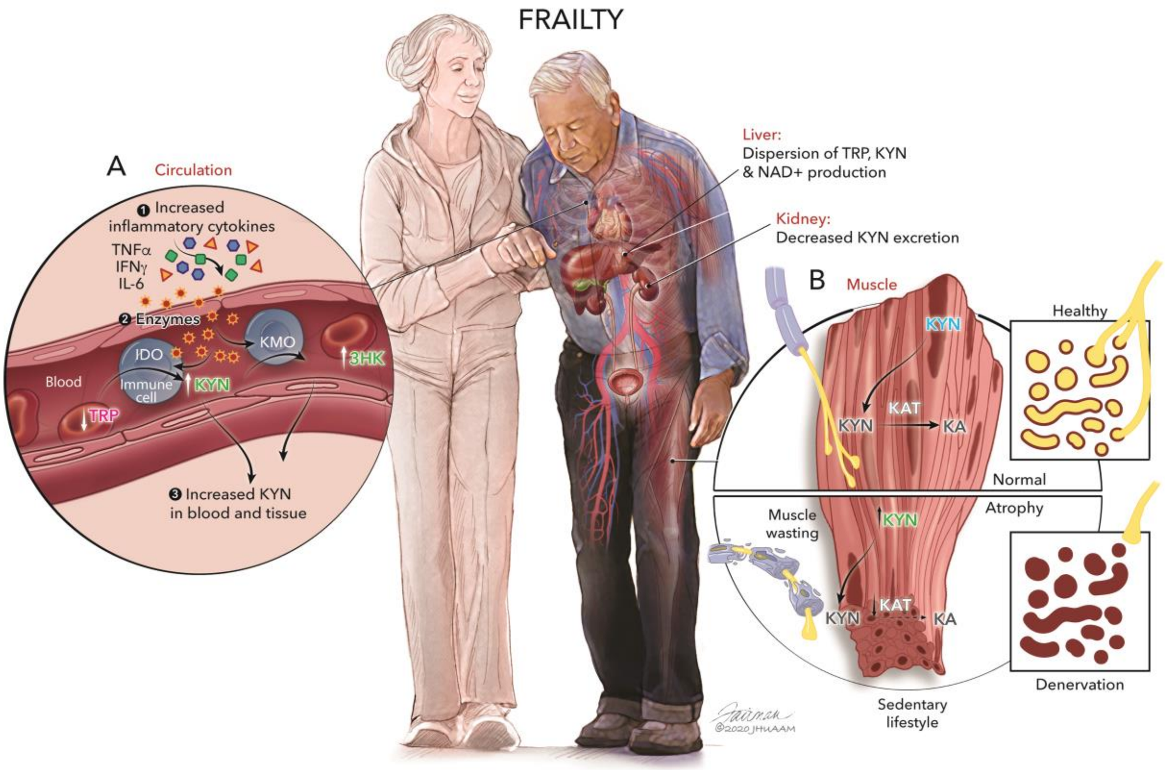

Supplemental Figure 3. Schematic summarizing the physiological connections between inflammation, kynurenines and neuromuscular integrity. (A) Increased levels of inflammation trigger IDO and KMO in immune cells to produce more kynurenines. (B) These kynurenines can damage neuromuscular junctions leading to decreased neuromuscular integrity.
